# Case of Complete Ossification of the Fœtal Head

**Published:** 1856-10

**Authors:** 


### A CASE OF COMPLETE OSSIFICATION OF THE FŒTAL HEAD.

BY PROF. ALLEN.

I was called in consultation by Dr. Haynes of this place, to see Mrs. M——, in protracted labor. There had been vigorous uterine action for twelve hours, the waters had been discharged, the head presenting in the first position. The relative capacities of the head and pelvis semed properly proportioned, yet there had been no advance of head for many hours. After making thorough exploration, no efficient cause for the tardy expulsion could be arrived at. There could no fontanellas be traced—the sutures were closely adpested, and indeed, a miniature *adult* head, was presenting. There could be no lapping of the bones, no diminution of size from compression, and cramotomy was determined upon. So firm was the asseous matter, that the performator could not be introduced by any safe force. Prof. Hughes, was then requested to see the case, and bring some instrument, by which the bone could be penetrated. By persevering drilling, and the use of a bone saw, the head was, at last, opened, the brain removed, the forceps applied, and the head, after some considerable effort, was brought into inferious passages and delivered. On examination, the sutures were found as firmly united as on the adult—the asseous matter almost flinty. The difficulty was, undoubtedly, in the non-compressibility of the head, as the proportions were such as would under the ordinary state of the fœtal head, have been entirely consistent with a natural and easy delivery.

